# Identification of viruses in Acute Lower Respiratory Infections (ALRI) in Lao People's Democratic Republic

**Published:** 2011-01-10

**Authors:** Anne-Charlotte Sentilhes, Vimatha Xaysitthideth, Sareth Rith, Somvay Ongkhamme, Thongchanh Sisouk, Darouny Phonekeo, Jean-Jacques Bernatas, Vincent Deubel, Philippe Buchy, Paul Brey, Phengta Vongphrachanh

**Affiliations:** 1 National Center for Laboratory and Epidemiology, Vientiane, Lao PDR; 2 Institut Pasteur in Laos, Vientiane, Lao PDR; 3 Institut Pasteur in Cambodia, Phnom Penh, Cambodia

## Background

Acute respiratory infections are a major cause of mortality and morbidity worldwide. Information on viral etiology in ALRI from Lao PDR is limited. The aim of the present study was to use Multiplex PCR/RT-PCR methods for the detection of the major respiratory viruses.

## Methods

Nasal/throat swab specimens were collected from patients enrolled in the ALRI surveillance programme within 2 hospitals, one in Vientiane Capital (Setthathirat Hospital) and the second one in Luang Prabang. From each sample, viral RNA was extracted and amplified by using 5 multiplex PCR/RT-PCR targeting 18 respiratory viruses.

## Results

Between 2009 and 2010, Multiplex PCR / RT-PCR detected respiratory viruses in 111 (54.7%) of 203 samples. Single virus was detected in 44.8% (91/203) and virus co-infections were observed in 9.9% (20/203). Rhinovirus (40.5%), human Respiratory Syncytial virus (hRSV; 27.9%), and Influenza A virus (9.0%) were the most frequently detected viruses. Adenovirus and human Metapneumovirus were detected in 8.1% and 6.3% of ALRI specimen, respectively. Influenza C virus and SARS-coronavirus were not detected during the study period. Children < 5 years represented 50% of patients identified.

## Conclusion

Our results provide new documentation about etiology of respiratory virus diseases in Lao People's Democratic Republic. In this context, multiplex PCR/RT-PCR offers a sensitive and reasonably priced diagnostic method for common respiratory viruses.

